# Light-Induced Energetic Decoupling as a Mechanism for Phycobilisome-Related Energy Dissipation in Red Algae: A Single Molecule Study

**DOI:** 10.1371/journal.pone.0003134

**Published:** 2008-09-04

**Authors:** Lu-Ning Liu, Abdalmohsen T. Elmalk, Thijs J. Aartsma, Jean-Claude Thomas, Gerda E. M. Lamers, Bai-Cheng Zhou, Yu-Zhong Zhang

**Affiliations:** 1 State Key Lab of Microbial Technology, Marine Biotechnology Research Center, Shandong University, Jinan, People's Republic of China; 2 Biophysics Department, Huygens Laboratory, Leiden University, Leiden, The Netherlands; 3 UMR 8186 CNRS & Ecole Normale Supérieure, Biologie Moléculaire des Organismes Photosynthétiques, Paris, France; 4 Institute of Biology, Leiden University, Leiden, The Netherlands; University of Oldenburg, Germany

## Abstract

**Background:**

Photosynthetic organisms have developed multiple protective mechanisms to prevent photodamage *in vivo* under high-light conditions. Cyanobacteria and red algae use phycobilisomes (PBsomes) as their major light-harvesting antennae complexes. The orange carotenoid protein in some cyanobacteria has been demonstrated to play roles in the photoprotective mechanism. The PBsome-itself-related energy dissipation mechanism is still unclear.

**Methodology/Principal Findings:**

Here, single-molecule spectroscopy is applied for the first time on the PBsomes of red alga *Porphyridium cruentum*, to detect the fluorescence emissions of phycoerythrins (PE) and PBsome core complex simultaneously, and the real-time detection could greatly characterize the fluorescence dynamics of individual PBsomes in response to intense light.

**Conclusions/Significance:**

Our data revealed that strong green-light can induce the fluorescence decrease of PBsome, as well as the fluorescence increase of PE at the first stage of photobleaching. It strongly indicated an energetic decoupling occurring between PE and its neighbor. The fluorescence of PE was subsequently observed to be decreased, showing that PE was photobleached when energy transfer in the PBsomes was disrupted. In contrast, the energetic decoupling was not observed in either the PBsomes fixed with glutaraldehyde, or the mutant PBsomes lacking B-PE and remaining b-PE. It was concluded that the energetic decoupling of the PBsomes occurs at the specific association between B-PE and b-PE within the PBsome rod. Assuming that the same process occurs also at the much lower physiological light intensities, such a decoupling process is proposed to be a strategy corresponding to PBsomes to prevent photodamage of the photosynthetic reaction centers. Finally, a novel photoprotective role of γ-subunit-containing PE in red algae was discussed.

## Introduction

Phycobilisomes (PBsomes) are the major light-harvesting antennae complexes in cyanobacteria and red algae. They are capable to absorb solar light and transfer energy to the chlorophylls (Chls) of photosynthetic reaction centers (RCs) with a high efficiency. PBsomes are supramolecular protein complexes made up of water-soluble phycobiliproteins (PBPs) and linker polypeptides [1]–[3]. PBPs are a distinctively colored group of disk-shaped macromolecular proteins bearing covalently attached open-chain tetrapyrroles, known as phycobilins, orderly assembled into PBsomes. In the absence of photosynthetic RCs, the PBsomes are highly fluorescent. Four spectral groups of PBPs are commonly identified: phycoerythrins (PEs), phycoerythrocyanins (PECs), phycocyanins (PCs) and allophycocyanins (APCs). Solar energy is absorbed by the chromophores of PEs (λ_max_ = 545∼565 nm) and transferred by nonradiative transfer in turn via PCs (λ_max_ = 620 nm), APCs (λ_max_ = 650 nm).

The conventional PBsomes in cyanobacteria, namely hemidiscoidal PBsomes, contain six peripheral rods and a core complex. The former is generally composed of peripheral PEs and inner PCs, and three parallel cylinders consisting APCs form the PBsome core complex. In contrast, hemiellipsoidal PBsomes in the unicellular red alga *Porphyridium cruentum* contain a large amount of PE molecules at the peripheral rod endings [4], [5]. In addition to R-phycocyanin (R-PC) and APC, two different spectral types of PEs were found in *P. cruentum*: B-phycoerythrin (B-PE) and b-phycoerythrin (b-PE) [6]. A specialized type of linker polypeptides, the chromophoric γ subunits, are responsible for the association of B-PE molecules in the rod elements, whereas they are absent in b-PEs [3], [7].

The evolution of PBPs, from APC to PC and to PE, is accompanied by the diversity and increasing number of chromophores [8]. The spectral diversity of different PBPs within PBsomes is assumed to extend the absorbance range (500–650 nm) of cyanobacteria and red algae, and offers a stepwise transfer of the trapped energy to the RCs. More specifically, the presence of PE in red algae, especially the γ-subunit-containing PE, could broaden the absorbance of red algae which allows increasing light-harvesting capacity.

The increased number of chromophores ensures higher light-harvesting yield of photosynthesis. Alternatively, it may be lethal if excess light in relation to the capacity of photosynthetic RCs absorbed by PBsomes in red algae is completely transferred to the RCs. In response to the excess excitation energy, a soluble orange carotenoid protein (OCP) has been demonstrated to be highly involved in the energy dissipation in cyanobacteria [9]–[11]. In contrast, the potential photoprotective response of PBsomes themselves with respect to excess energy is less understood.

The spectroscopy of single molecules or complexes has the great advantage to avoid the ensemble averaging encountered in bulk spectroscopy. Molecular dynamics, for instance molecular conformational changes, energy transfer and photobleaching, can be studied by measuring the real-time fluorescence spectrum at single molecule sensitivity [12]. Single-molecule techniques have been extensively applied in photosynthetic systems and provided new insights into the photophysical processes and functional properties of photosynthetic chromophore-protein complexes [13]–[18]. Effects of photobleaching and interprotein energy transfer of single PBPs have been previously reported, for systems such as B-PE [19], monomers of PEC [20], [21] and APC trimers [22]. However, spectral properties of intact single PBsome complex are still enigmatic.

Recently a novel approach was explored to monitor single-molecule fluorescence spectra using an Amici prism as a dispersion element [23], [24]. It provides the possibility to investigate real-time fluorescent emission dynamics of PBsomes as well as the energy transfer between various PBsome components. In the present work, we applied single-molecule spectroscopy to investigate the PBsomes from *P. cruentum* at room temperature. Green-light-induced photobleaching of single PBsome was demonstrated to involve an energetic decoupling of B-PE within the PBsome rod, serving as a novel pathway of energy dispassion. Our results provide insights into the photobleaching dynamics of PBsomes in red algae, and their photoprotective roles in response to excess light energy.

## Results

### Fluorescence photobleaching of single PBsome

**Figure 1:**
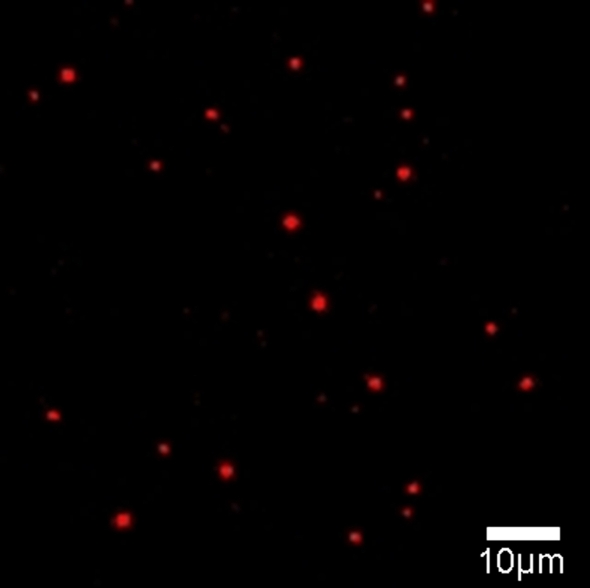
Fluorescence image (82×82 µm) of individual PBsomes at room temperature. Excitation wavelength is 532 nm.

Green (532 nm) and red (639 nm) lasers were utilized in this work for high-light illumination. Based on the ensemble absorption spectrum of PBsomes from *P. cruentum*, the 532 nm light is absorbed predominantly by PE, allowing to investigate the whole energy transfer process within intact PBsomes, from PE to R-PC, APC and finally to the PBsome terminal emitters, L_CM_ and α^APB^ [25]. Figure 1 shows the raw fluorescence image of individual PBsomes excited at 532 nm (260 W·cm^−2^). PBsome complexes were immobilized on the glass slide with polyvinyl alcohol (PVA) solution (solublized with 0.75 M phosphate buffer, pH 7.0). Due to the diluted concentration (0.5 µg·ml^−1^), most of the observed intensity spots represent single PBsomes. A good signal-to-noise ratio of the fluorescence emission was obtained due to the strong fluorescence of PBsome supramolecular complex which carries up to 2000 bilin chromophores under highly sensitive detection.

**Figure 2:**
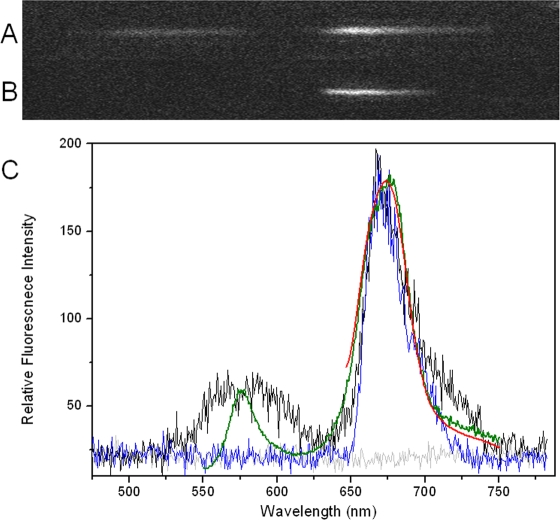
Fluorescence emission image of single PBsome at room temperature. Images were obtained in wide-field microscopy dispersed by prism in front of CCD camera. Single PBsome was imaged as two bands when excited at 532 nm (A), and a single fluorescence band was recorded when excited at 639 nm (B). C, fluorescence emission spectra of single PBsome excited at 532 nm (black) and 639 nm (blue). Two fluorescence emission bands of resemble PBsomes were observed at 580 nm and 674 nm when excited at 532 nm, and only 674 nm emission peak was visible when excited at 639 nm. Background is presented with light gray. Room-temperature fluorescence emission spectra of isolated PBsome solution (0.75 M phosphate buffer, pH 7.0) were recorded when excited at 532 nm (green) and 639 nm (red).

After dispersion by an Amici prism, two discrete fluorescence bands were recorded simultaneously when single PBsome was excited at 532 nm (Figure 2A). Full fluorescence emission spectrum of single PBsome was generated by integrating the total fluorescence intensities within a selected region after subtracting the background. Figure 2B shows the fluorescence image of single PBsome excited with 639 nm. As depicted in Figure 2C, a major fluorescence emission from the PBsome core (674 nm) and a minor fluorescence emission band of PE (576 nm) were observed. We recorded fluorescence spectra of 200 single PBsome complexes acquired with a 400 ms integration time per spectrum. 80% of them (162 single PBsomes) present typical fluorescence of PBsomes. Spectral variation was observed in other 20% PBsomes, showing decreased fluorescence and a higher ratio of PE/PBsome core fluorescence. It is assumed that the photobleaching of a few PBsomes has taken place under laser illumination in the process of seeking targeting samples. Excitation energy at 639 nm is only absorbed by APC complexes. Therefore, the fluorescence emission of PBsome complex was detected as only a single fluorescence band attributed to the PBsome core (Figures 2B and 2C). These are consistent with the bulk fluorescence spectra (Figure 2C).

**Figure 3:**
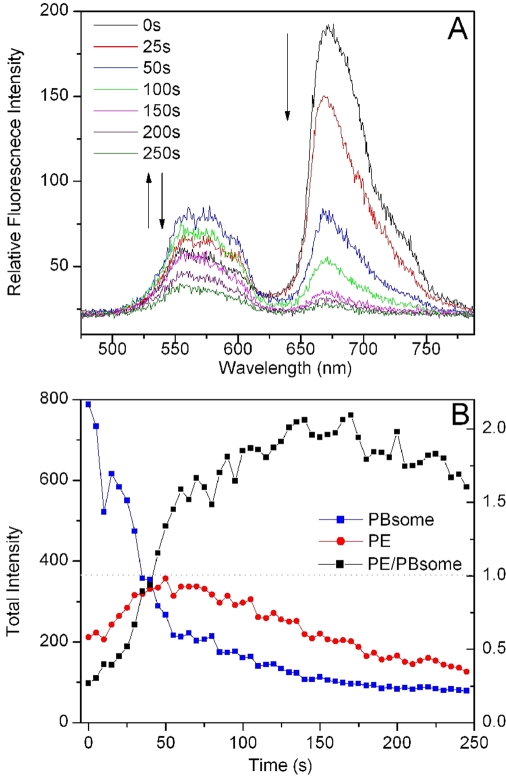
Fluorescence photobleaching of single PBsome under 532 nm illumination at 260 W·cm^−2^ during 250 seconds. Integration time is 400 ms. A, fluorescence spectral profiles of single PBsome during photobleaching. Arrows represent the fluorescence changes of PE and PBsome core. B, total fluorescence intensities of PE (red) and PBsome core (blue) fluorescence emission of single PBsome as well as the ratio of fluorescence intensities of PE/PBsome core (black) as a function of time.

We further investigated the fluorescence photobleaching of individual PBsome complexes. As shown in Figure 3A, under illumination with 532 nm laser at 260 W·cm^−2^, PBsome core fluorescence exhibited successive reduction of fluorescence intensity with a minor blue-shift. This implies that intense green-light enables to induce the photobleaching of intact PBsome. Surprisingly, PE fluorescence did not present the same photobleaching behavior as PBsome core fluorescence. Instead, an increase of PE fluorescence intensity at the first seconds of illumination was observed, followed by the subsequent reduction. This is clearly evident in the fluorescence intensity traces of two emission bands as a function of time (Figure 3B). It shows a 1.6-fold fluorescence increase of PE emission at the first stage of illumination and a decrease after 50 seconds. These two distinct stages of PBsome are further revealed by investigating the ratio of PE to PBsome core fluorescence. As seen in Figure 3B, the ratio of the fluorescence intensities of PE to PBsome core initially rises drastically above the value of 1.0, due to the combination effects of the increase of PE emission and rapid reduction of PBsome core emission; afterward it alters insignificantly, indicating that at the late stage both emissions are bleached with the relatively equivalent rates. The real-time spectral dispersion provides the opportunity to detect the fluorescence dynamics of PBsome which is highly correlated with changes of energy migration. In view of the compositional heterogeneity and energy transfer between PBsome components, the photobleaching profile of single PBsome is different from those of individual PBPs observed before [19]–[22].

### Power-dependence of single PBsomes during photobleaching

**Figure 4:**
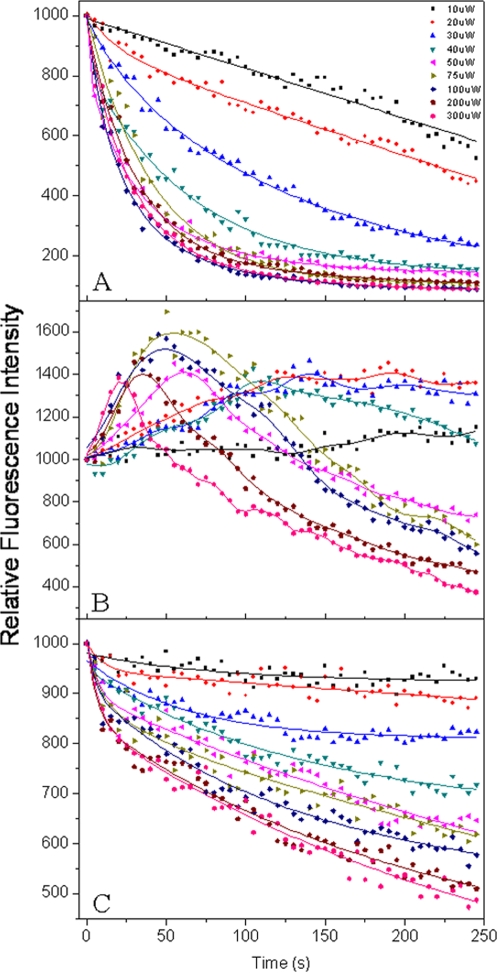
Power-dependence of fluorescence photobleaching of single PBsome. Laser power is given by W·cm^−2^. A, normalized PBsome fluorescence intensity time courses over the laser power range from 35 to 1050 W·cm^−2^. Excitation wavelength is 532 nm. B, normalized PE fluorescence intensity time courses over the laser power range from 35 to 1050 W·cm^−2^. Excitation wavelength is 532 nm. C, normalized PBsome core fluorescence intensity time courses over the laser power range from 35 to 1050 W·cm^−2^. Excitation wavelength is 639 nm which can only excite APC.

The light intensity encountered in single-molecule spectroscopy is many orders of magnitude higher than natural one, and the photobleaching under high-light condition occurs, on different time-scales, with the chromophoric systems. We explored the effect of light intensities on the photobleaching behaviors of single PBsome complex (Figure 4). Figure 4A shows the effects of green laser (532 nm) with different laser powers (35 to 1050 W·cm^−2^) on the fluorescence emission from PBsome cores. The bleaching amplitude of PBsome core emission was strongly depending on the laser power up to 140 W·cm^−2^. Light intensities lower than 140 W·cm^−2^ did not induce maximum fluorescence quenching within 250 seconds. Above 140 W·cm^−2^, the photobleaching profiles appeared to be nearly indistinguishable and more than 80% photobleaching was detected. Figure 4B presents power-dependence of PE emission with excitation at 532 nm. With the increase of laser power, a significant rise of PE emission intensity was observed and was also shown to be closely correlated to the laser power. The maximum intensity was detected at 260 W·cm^−2^. The PE intensity traces reflect competition between an increase of fluorescence intensity and photobleaching. Above 260 W·cm^−2^, the photobleaching start to dominate, limiting the maxima incline of fluorescence intensities. Furthermore, the maximum emission intensity presents a progressive blue-shift with the increase of laser power, indicating that more intense light can result in a faster increase of PE emission. Figure 4C shows the power dependence of PBsome core emission with excitation at 639 nm. Since only APCs can be excited at 639 nm, the power-dependent behavior of PBsome core emission may be compared with the PBsome core fluorescence when excited at 532 nm, to provide more insights into energy transfer of intact PBsome. It was found that the fluorescence of PBsome core complex is unable to be completely bleached (at most up to 50%) in 250 seconds. With absorbance of PBsomes at 532 nm being 6-fold higher than that at 639 nm (see absorption spectrum shown below in FLUORESCENCE PHOTOBLEACHING OF MUTANT PBSOME), 1050 W·cm^−2^ red laser power is comparable to 175 W·cm^−2^ green laser power upon excitation the PBsome complex. 1050 W·cm^−2^ red laser induced 50% of photobleaching, whereas 175 W·cm^−2^ green laser resulted in as low as 80% of photobleaching, suggesting that the fluorescence loss of PBsome core when excited at 532 nm is composed of not only the relevant photobleaching of PBsome core emission, but the energy transfer disturbance which is correlated to the increase of PE emission.

### Green laser induces an energetic decoupling of the PBsome

The possibilities for the fluorescence increase of PE were suggested. First, fast energy transfer from PE to PBsome core allows the PE complexes to be less bleached than PBsome terminal emitters. When down-stream energy acceptors are bleached, excess energy captured by peripheral PE molecules is dissipated from PE. Another possibility is the decoupling of PE from energy transfer path in PBsome. Excess energy may increase the rate of bleaching of PE which exists as an isolated individual energy acceptor after the energetic decoupling, and result in further decrease of PE emission. In addition, it is also possible that energy transfer within the PBsomes is much decreased due to the photodamage without physical decoupling of the complex [26].

**Figure 5:**
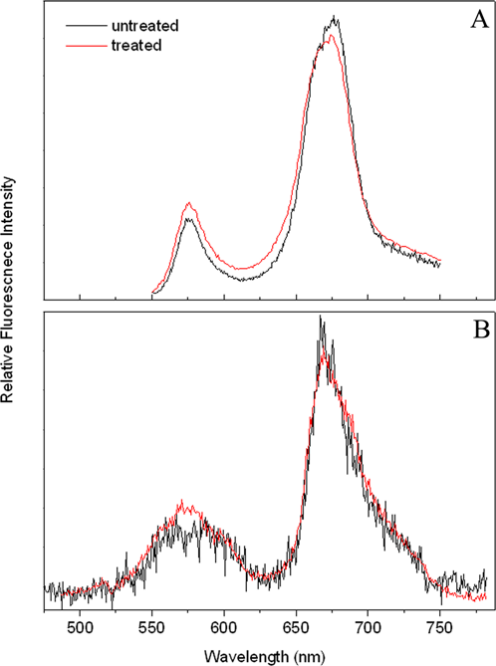
Comparison of the fluorescence properties of PBsomes treated and untreated with GA. Excitation wavelength is 532 nm. A, ensemble fluorescence spectra; B, single-molecule fluorescence spectra.

**Figure 6:**
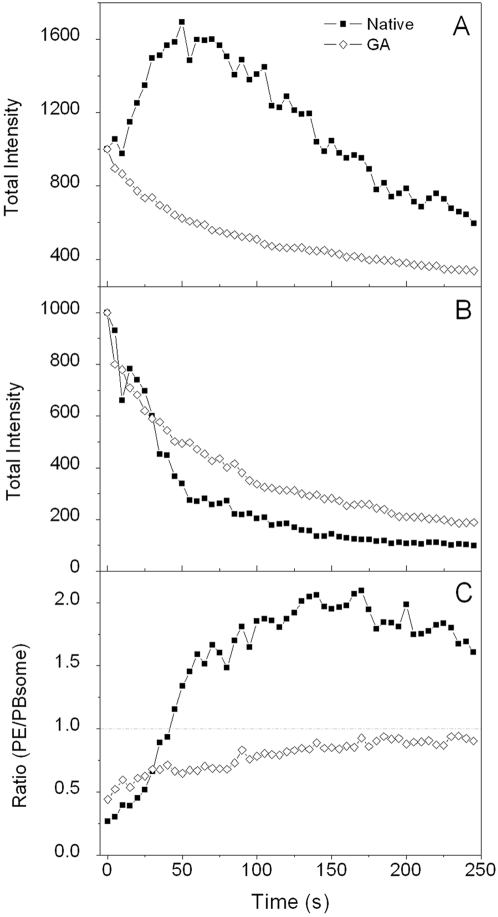
Fluorescence photobleaching of PBsome treated and untreated with GA under illumination power of 260 W·cm^−2^. A, time course of the photobleaching of PE emission after normalization; B, time course of the photobleaching of PBsome core emission after normalization; C, time course of the ratio of fluorescence intensities of PE/PBsome core.

To further reveal the mechanism involved in the fluorescence increase of PE, we investigated single PBsome complex pre-treated with protein cross-linking agent glutaraldehyde (GA). GA has been commonly used to stabilize PBsome conformation [5], [27], [28]. Under the treatment of GA (1%, v/v), the PBsome is assumed to retain conformational and functional intact. This is corroborated by the fact that bulk (Figure 5A) and single-molecule emission spectra (Figure 5B) before and after GA treatment are identical. Figure 6A shows time courses of the total intensity of PE emission in single PBsome untreated and pre-treated with GA. In contrast to the photobleaching behavior of untreated PBsome, no increase of PE emission was observed after fixation with GA, which indicates that the increase of PE emission observed in the photobleaching of untreated PBsome may be attributed to the energetic decoupling of PE in the PBsome. In Figure 6B, the photobleaching percentage of PBsome core emission treated with GA is found to be 10% less than that of untreated PBsome. Such a difference is assumed to be attributed to the energetic decoupling of PE molecules. Figure 6C presents the distinct ratios of PE to PBsome core fluorescence intensity in these two conditions. Untreated PBsome exhibited a drastic incline of the ratio up to the value of 2.0, which suggests that after bleaching PE fluorescence is more pronounced than that of the PBsome core. In contrast, the ratio of fluorescence intensity of the PBsome with GA treatment was observed to be less increased, constantly lower than the value of 1.0. Our data revealed that green-laser-induced energetic decoupling might involve changes of the protein-protein associations within PBsome.

### Fluorescence photobleaching of mutant PBsome

**Figure 7:**
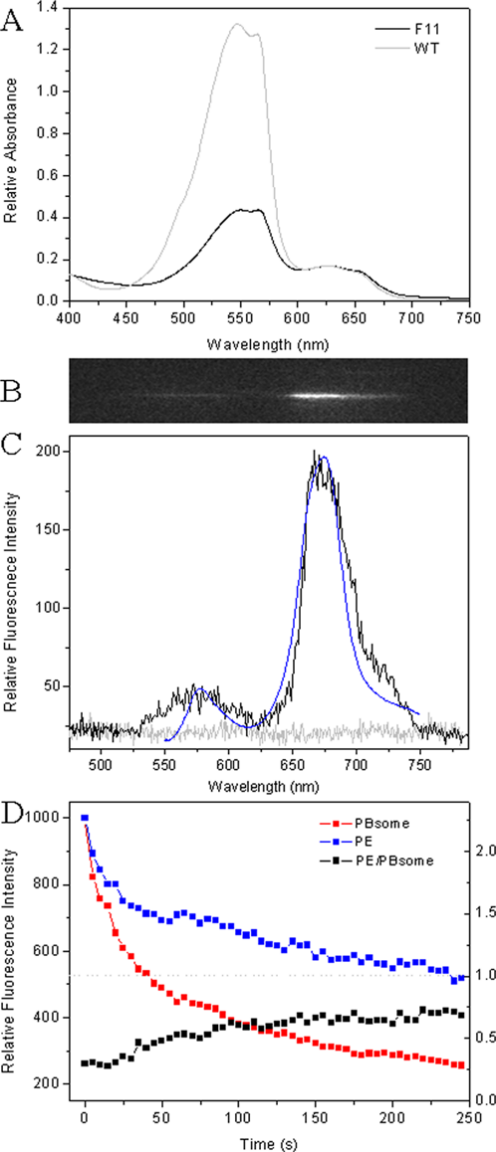
Single-molecule properties of PBsome from mutant *P. cruentum* F11. A, absorbance of ensemble PBsome from WT and mutant strain. A_532_ of F11 PBS is as low as 35% than WT PBsome, so laser power performed on F11 PBsome is 700 W·cm^−2^. B, fluorescence emission image of single F11 PBsome at room temperature obtained in wide-field microscopy dispersed by the Amici prism in front of CCD camera. C, single PBsome was imaged as two fluorescence bands when excited at 532 nm (black). Background is presented with light gray. Room-temperature fluorescence emission spectra of isolated mutant PBsome (0.75 M phosphate buffer, pH 7.0) were recorded when excited at 532 nm (blue). D, total intensities of PE (▴) and PBsome core (▪) emission of single PBsome as well as their ratio (□) as a function of time.

It is noteworthy to mention that the light-induced decoupling of PE in single PBsome can only result in no more than 2 times of fluorescence increase and energy transfer within the complex can still take place. It apparently implies two discrete types of PEs: one group of PEs are involved in the light-induced decoupling, and another group still have a solid association in the PBsome rods to perform energy flow. This is reminiscent of previous observation that both B-PE and b-PE were found in the rod of *P. cruentum* PBsomes [6]. Their difference was further indicated to be the presence of γ peptide in B-PE but not in b-PE [7]. The mutant strain F11 of *P. cruentum* constructed was characterized to contain lower level of PE content in light of the complete deficiency of γ subunits, the B-PE-associated chromophoric linker polypeptides, revealed by LiDS-PAGE (lithium dodecyl sulfate polyacrylamide gel electrophoresis) experiment [29], [30]. Absorption spectra of ensemble PBsomes from wild-type (WT) and F11 *P. cruentum* show that one third of PE is retained in the mutant PBsome compared to WT PBsome (Figure 7A). It is most likely that the remaining are b-PE molecules which assemble with neighboring R-PC by linker polypeptides which are different from the γ subunits. The smaller dimension of mutant PBsome because of less PE content is also confirmed by our TEM result (data not shown).

As a comparative investigation, we examined the fluorescence emission properties of single PBsome prepared from mutant F11. In terms of the reduced absorbance at 532 nm in this mutant, green laser of 700 W·cm^−2^ was applied on the F11 PBsome compared to 260 W·cm^−2^ on WT PBsome in order to obtain the identical excitation. Figure 7B shows a dispersed fluorescence emission of single PBsome from F11 excited at 532 nm green laser. Two separated fluorescence bands are detected, reminiscent of the fluorescence property of WT PBsome. In addition to the identical emission positions, we found that the PE fluorescence of mutant PBsome is weaker than that of WT PBsome, which probably suggests a higher efficiency of energy transfer in mutant PBsome (Figure 7C). It may imply the flexible assembly between B-PE and b-PE in WT PBsomes, as well as the relatively solid interaction between b-PE and neighboring R-PC hexamer. During the process of photobleaching, both PE and PBsome core emissions of F11 PBsome are quenched in parallel (Figure 7D). The intensity ratios of b-PE to PBsome core fluorescence are relatively constant, lower than the value of 1.0. Unlike WT PBsome, the increase of PE emission intensity during the bleaching was not seen, suggesting that B-PE molecules which are located at the peripheral side of WT PBsome rods are predominately responsible for the energetic decoupling of PBsome, whereas b-PE is probably not involved.

## Discussion

The strong fluorescence of the PBsomes, due to the abundance of chromophores carried in the PBsomes, is shown to be an advantage for the sensitive detection of individual complexes. We applied fluorescence detection at the single-molecule level to investigate, for the first time, the fluorescence of the PBsomes in *P. cruentum* dispersed through an Amici prism. The real-time fluorescence detection is able to reveal the spectral dynamics and energy transfer of the chromophore-protein complexes.

The results allow us to better characterize the effects of intense light on the PBsomes. It is demonstrated that strong green-light can induce the photobleaching of the PBsomes, as shown by the drastic decline of PBsome core fluorescence. More importantly, a great proportion of excitation energy, surprisingly, is found to be dissipated in the form of PE fluorescence. Our comparative results, which show that such an increase is absent in the photobleaching of GA-treated PBsome, verified the green-light-induced energetic decoupling of PE in the PBsome.

The PBsome rods in cyanobacteria comprise in general one or up to two types of PBPs, whereas those in red alga *P. cruentum* contain in turn B-PE that carries γ-subunit, b-PE that does not carries γ-subunit, and R-PC from the periphery to the interior of the rod. Therefore, the PBsomes of *P. cruentum* is an ideal model to study the energy transfer pathway. The different behaviors of PE fluorescence in WT and mutant experiments presented in this work strongly indicate the distinct contributions of B-PE and b-PE in the energy transfer of PBsomes.

**Figure 8:**
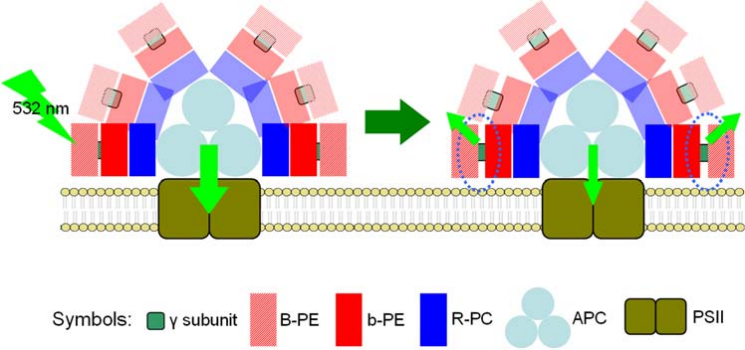
Schematic model of light-induced energetic decoupling of PBsomes. Arrows represent the fluorescence emissions from PBsome components during photobleaching. Circles present the potential decoupling sites within PBsome.

On the basis of our observations, a schematic model of light-induced energetic decoupling of PBsome was proposed, as depicted in Figure 8. It is characterized that B-PE and b-PE are coupled face-to-face at the periphery of PBsome rod. The outer B-PE presents potentially a flexible energetic association with the inner b-PE with the assistance of the γ subunit of B-PE. The photobleaching of PBsome is accompanied by the energetic decoupling which preferentially occurs in the b-PE - B-PE interaction site. It is presumably that the γ subunits, the specific linker polypeptides which carry phycobilins, might play an important role in the energetic decoupling of PBsomes in *P. cruentum*.

On the other hand, assuming that the same process occurs also at the much lower physiological light intensities, our observations have important implications on the photoprotection function of PBsomes. So far, the mechanism of the antenna-related photoprotection of OCP in cyanobacteria has been elucidated [9]–[11]. The general concept is that PBsome can not dissipate excess absorbed energy without assistance [31]–[33]. Such an energetic decoupling of PBsomes with respect to intense light may presumably allow excess photon energy from PE to photosynthetic RCs to be modulated to minimize the risk of chlorophyll photooxidation. This is corroborated with the findings about high-light-induced reorganization [34] and photodegradation of PBsome [35]. The light-induced conformational altering of PBsome was also found in UV-treated PBsome from which a PE fluorescence increase has been observed, arising from the disassembly of PBsome components [36].

The photoprotection role of PE in the PBsomes of cyanobacteria on dissipating excess light energy to prevent the photodamage of RCs has been previously interpreted [37]. Here, despite the high involvement of PE in the light-induced energetic decoupling of PBsomes, we further found specifically that the different roles of B-PE that carries chromophoric γ-subunit and b-PE that lacks γ-subunit in the photoprotective mechanism of red algae. In addition to the roles in PBPs assembly and energy migration, the chromophoric γ subunit is preferentially sensitive to the intense light, and probably functions in the photoprotection of PBsome. As a primitive unicellular red alga, *P. cruentum* contains the specific γ subunits and colorless linker polypeptides to assemble B-PE and b-PE hexamer complexes, respectively, in the PBsome rods. The evolution of cyanobacteria and red algae is revealed to be accompanied by the evolution of PBPs [8]. The chromophore variety and increasing number extend the absorbance spectrum and enhance the absorption capacity, enabling the photosynthetic organisms to survive in various environments. Furthermore, our data provide new insights into the biological roles of the chromophore variety: the spectral variety of PBPs in intact PBsomes and the appearance of chromophoric γ subunit may generate a multi-step photoprotection to effectively prevent photodamage of photosynthetic RCs in response to excess excitation energy *in vivo*.

## Materials and Methods

### Sample separation

*Porphyridium cruentum* wild-type and mutant F11 strain (UTEX 637) was grown in an artificial sea water medium [38]. Flasks were supplied with 3% CO_2_ in air through a plug of sterile cotton at a constant temperature of 20°C. Cultures were illuminated continuously with light provided by daylight fluorescent lamps at 6 W·m^−2^. The *P. cruentum* F11 was constructed before [29] and was described with the deficiency of γ subunit element as examined by LiDS-PAGE [30]. Intact PBsomes were separated following the previous protocol [5].

### Spectral analysis

Absorbance spectra were recorded with UV-160A spectrophotometer (Shimadzu, Japan). Room-temperature fluorescence spectra were measured with LS 55 Luminescence Spectrometer (Perkin-Elmer Instruments, USA).

### Single-molecule experimental setup

PBsomes were dissolved in 0.75 M phosphate buffer with 1% polyvinyl alcohol (PVA) to a protein concentration of 0.5 µg·ml^−1^, and then samples were spincoated on the freshly cleaned coverglass. For GA fixation, PBsomes were diluted with above PVA buffer containing GA with a final concentration of 1% (v/v).

The single-molecule fluorescence setup is based on an Axiovert S100TV inverted microscope with epifluorescent detection. The power density at the sample was 35–1050 W·cm^−2^. The light from the laser sources, Nd∶YAG (λ = 532 nm) and solid state laser (λ = 639 nm), was passed through a set of optical components (to control intensity and polarization) and a lens which is changing the type of illumination, reflected by a dichroic mirror (centered at 550 nm/660 nm), and focused on the sample by a high numerical aperture water-immersion objective (60×, numerical aperture 1.2, Olympus). Fluorescence emission from excited molecules was collected with the same objective, filtered after the dichroic mirror with an additional notch filter to reject the remaining scattered laser light and Raman scattered light. Fluorescence emission was dispersed by an Amici prism and detected with front-illuminated charge-coupled device (CCD) camera (Cascade 650, Photometrics). The integration time used for the spectra acquisition was 0.3 seconds.
